# Long-term results after anterior cruciate ligament reconstruction using patellar tendon versus hamstring tendon autograft with a minimum follow-up of 10 years—a systematic review

**DOI:** 10.1007/s00402-022-04687-9

**Published:** 2022-11-28

**Authors:** V. D. Sollberger, A. Korthaus, A. Barg, G. Pagenstert

**Affiliations:** 1 CLARAHOF Clinic of Orthopaedic Surgery, University of Basel, Clarahofweg 19a, 4058 Basel, Switzerland; 2 Department of Trauma and Orthopaedic Surgery, University Medical Center Hamburg-Eppendorf, Germany, Martinistr. 52, 20251 Hamburg, Germany; 3 Department of Trauma Surgery, Orthopaedics and Sports Traumatology, BG Hospital Hamburg, Bergedorfer Str. 10, 21033 Hamburg, Germany

**Keywords:** Anterior cruciate ligament reconstruction, Anterior cruciate ligament injury, Patellar tendon, Hamstring tendon, Knee instability, Osteoarthritis, Long-term outcome

## Abstract

**Introduction:**

A lot of research addresses superiority of the two commonly used autografts bone-patellar tendon-bone (BPTB) and hamstring tendon for anterior cruciate ligament (ACL) reconstruction, without getting to consensus. While there are numerous studies and reviews on short- to mid-term follow-up, not much literature is available on long-term follow-up. As patients suffering ACL injuries are often of young age and high athletic activity, it is crucial to have the best evidence possible for graft choice to minimize consequences, like osteoarthritis later on.

**Materials and methods:**

A search of the online databases, PubMed and Embase, was carried out last on 31st March 2022 for studies comparing BPTB and hamstring tendon (HT) autografts for ACL reconstruction in human patients with a minimum follow-up of 10 years. The methodological quality of each study has been evaluated using the modified Coleman Methodology Score. Results on the three variables patient-oriented outcomes, clinical testing and measurements and radiographic outcomes were gathered and are presented in this review.

**Results:**

Of 1299 records found, nine studies with a total of 1833 patients were identified and included in this systematic review. The methodological quality of the studies ranged from a Coleman Score of 63–88. Many studies reported no or only few statistically significant differences. Significant results in favour of BPTB were found for activity levels and for instrumented laxity testing with the KT-1000 arthrometer. Better outcomes for HT were found in IKDC-SKF, the KOOS, donor site morbidity, pivot shift test, radiographic osteoarthritis (IKDC C or D) and contralateral ACL rupture. No studies presented significant differences in terms of Lysholm Score or Tegner Activity Score, Lachman test, single-legged hop test, deficits in range of motion, osteoarthritis using the Kellgren and Lawrence classification or graft rupture.

**Conclusion:**

We cannot recommend one graft to be superior, since both grafts show disadvantages in the long-term follow-up. Considering the limitation of our systematic review of no quantitative analysis, we cannot draw further conclusions from the many insignificant results presented by individual studies.

Level of evidence: IV.

## Introduction

Anterior cruciate ligament (ACL) tears have a major impact on the individual as well as the society. Injuries of the ACL are common and occur often in the younger population. A meta-analysis including 4108 patients shows that 10 years after ACL reconstruction around 20% and 20 years after ACL reconstruction around 50% of the patients suffer from osteoarthritis (OA) [11]. This highlights the importance of the ACL plastic and thus also the correct graft selection.

For reconstruction of the ACL different types of auto- and allograft are being used. While 8–12% of transplants were allografts between 2006 and 2012, they are currently only used for specific indications. In the past decades, a change in the type of autograft transplants used has been observed. While in 1992, bone-patellar tendon-bone (BPTB) represented the largest proportion of grafts with 90%, hamstring tendons gained importance over time due to less side effects like anterior knee pain and represented the most used graft in 2010. During this time, the quadriceps tendon (QT) gained importance so that it represented 10% of the tendons used in 2020. In comparison, the hamstring tendon (HT) was used as a graft in half of the ACL reconstructions and the BPTB only in 1/3 of the cases [3].

There are numerous studies comparing BTPB and hamstring tendon (HT) in short- to mid-term outcomes [1, 4, 7, 13, 16, 23, 28, 44]. However, there are only a few studies investigating the long-term outcome and there is no consensus on which one should be the graft of choice [35, 44].

Keays et al. showed that among other factors like meniscectomy, chondral damage and low quadriceps-to-hamstring strength ratios, the choice of graft for ACL reconstruction is a significant predictor for the development of OA [21].

In a 2011 Cochran review by Mohtadi et al., no superiority of BPTB or HT over each other could be established at 2-year follow-up [30]. Patients who underwent ACL surgery with autologous BPTB showed a more stable knee in the clinical examination after 2 years compared to autologous HT, but also suffered more from anterior knee pain [30].

In studies with a follow-up of 6, 7, and 10 years has been shown that ACL reconstruction with BPTB leads to significantly higher incidence of OA [20, 34, 37].

On the other hand, large registry study from Scandinavia and Denmark showed that patients with BPTB reconstruction have a statistically significant lower risk of revision [15, 36].

To help resolve the controversy between ACL reconstructions with BPTB and HT in the long-term outcomes, this systematic review analyses studies comparing these two autografts with a follow-up of a minimum of 10 years. The focus of this systematic review lies on the three variables: patient-oriented outcome, radiographic outcome and clinical testing and measurement.

## Materials and methods

The PRISMA (Preferred Reporting Items for Systematic reviews and Meta-Analyses) statement and the PRISMA 2020 Explanation and Elaboration Document were used as guidance for our systematic review and literature [32, 33]. The study was also registered at prospero under CRD42022310607.

### Search strategy

The medical databases, Embase and PubMed, were searched from inception through 31st of March 2022. The following search term was used: ((anterior AND cruciate) OR acl) AND (reconstruction OR surgery OR repair) AND (patella* OR bptb) AND (hamstring* OR semitendinosus OR gracilis). Additionally, we screened reference lists of the literature reviews on the same or similar topic for potential studies we missed in our systematic search of the databases but could not identify any new ones [2, 6, 10, 17, 19, 27, 29, 35, 44].

### Study selection

The literature selection obtained by the search term was first presorted using the abstract. If the suitability was unclear from the title and abstract, the full text of the article was obtained and checked for suitability. Studies were included that met the following criteria: comparison of ACL reconstruction using both HT and BPTB graft types in human patients, reporting on at least one of the three outcome variables (patient-oriented outcome, clinical tests and measurements of laxity as well as function, radiological outcome), and a follow-up period of at least 10 years. It was decided not to include studies with quadriceps tendon as graft because of the very limited number of long-term studies.

**Fig. 1:**
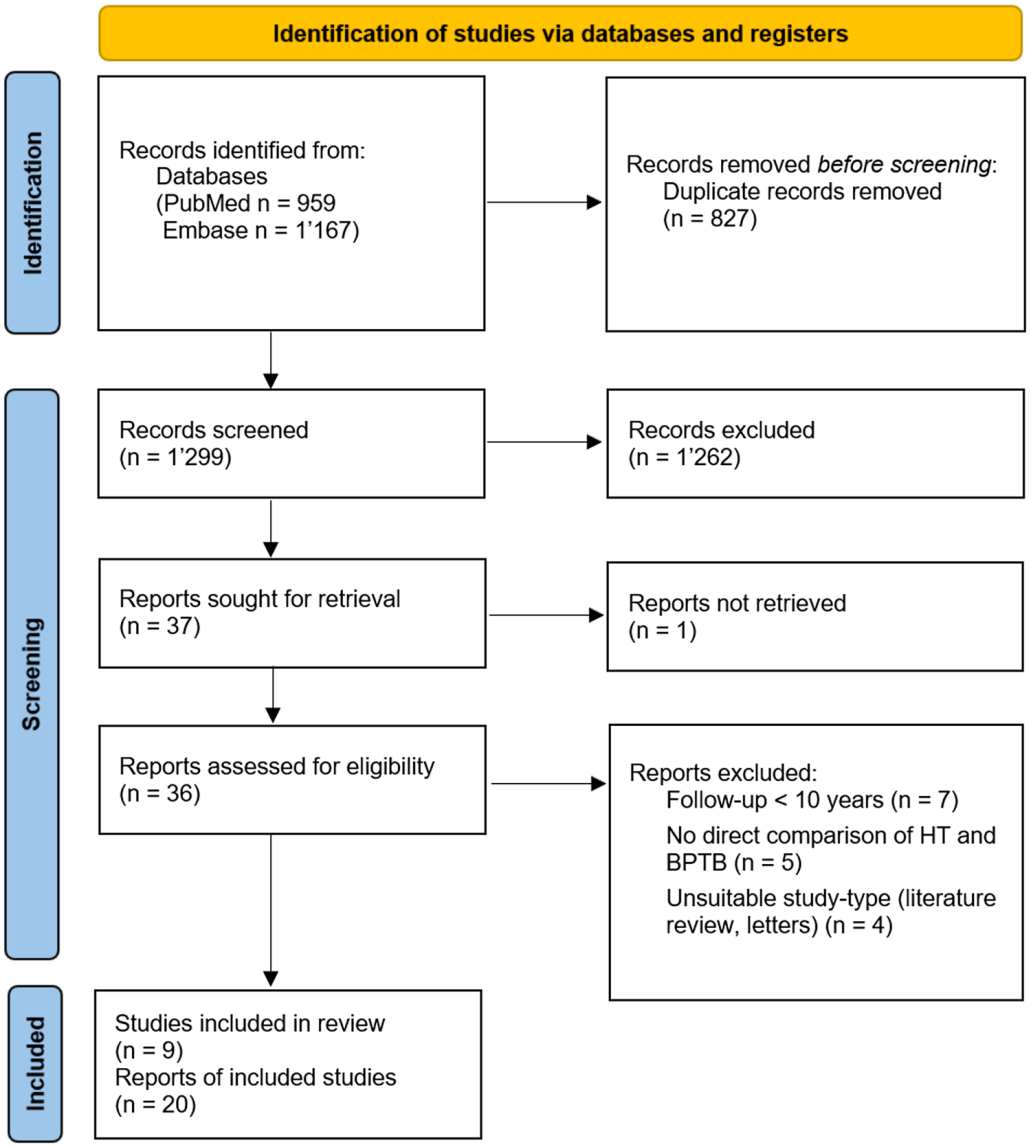
PRISMA (Preferred Reporting Items for Systematic Reviews and Meta-Analyses) flow diagram showing the selection process of studies included in this systematic review

Due to the following exclusion criteria, we had to exclude studies from our review: minimum duration of follow-up of less than 10 years, full text not available or in a language other than English, German, Spanish or French, literature reviews or letters, studies with exclusively paediatric patients, studies on cadavers, animals or biomechanical in vitro studies. In addition, if there were multiple reports based on the same collective, the one with the longest follow-up was included and the others were excluded (Fig. 1).

### Data extraction

The following data were extracted from the full text of all included studies: year of publication, study type, single centre or multicentre, duration of follow-up, number of patients in the BPTB and the HT group, surgical technique, patient-oriented outcomes (Lysholm score, IKDC subjective knee form, KOOS, Tegner Activity Score, other measurements of activity level, kneeling pain, anterior knee pain, knee walking test), radiographic outcomes (Kellgren and Lawrence Score, IKDC), clinical tests (Lachman test, pivot shift test, KT-1000 arthrometer, single-legged hop test, extension and flexion deficit) and graft rupture rates and rates of contralateral ACL rupture.

Two authors will review the title and abstracts of each article identified in the literature search. When eligibility is unclear from the title and abstract, the article’s full text was obtained and evaluated for eligibility. The whole process of study selection and data extraction has been done by two independent authors. Any disagreements will be resolved by consensus discussion between the two independent reviewers. A third author will be consulted if the debate cannot be resolved.

### Study quality assessment

To assess each study included in this review for its quality of methodology a modified version of the Coleman Methodology Score has been used. The Coleman Methodology Score has been developed to assess the methodology of studies reporting surgical outcomes. It consists of a Part A with seven items (study size, mean duration of follow-up, number of different surgical procedures used, type of study, diagnostic certainty, description of surgical procedure and postoperative rehabilitation) and a Part B with three items (outcome criteria, procedure for assessing outcomes and description of subject selection process) and the result is reported as a score from 0 to 100 [12]. The modification of the original Coleman Methodology Score has been made by adapting the values for the first item, study size, as shown in Table 6 in the “Results” section.

## Results

The search of PubMed and Embase with the above-mentioned search terms led us to 959 records from PubMed and 1167 records from Embase. After an additional screening of the reference lists and exclusion of duplicates, a total of 1299 records could be achieved. 1261 studies were excluded because they met the above exclusion criteria. Of the remaining 34 reports, we screened the full text and identified nine studies with a total of 1833 analysed patients, that met the eligibility criteria determined for this systematic review (Fig. 1).

### Study characteristics

The main characteristics of the nine studies included in this systematic review are presented in Table 1 in alphabetical order [18].

**Table 1:** Characteristics of the included studies

Study	Year	Journal	Study design	LoE	Coleman methodology score	Single or multicenter	Initial number of patients total (PT, HT)	Number of patients at final follow-up total (PT, HT)	Age at surgery (y) Mean (range)	Duration of follow-up (y) Mean (range)
Barenius et al. [5]	2014	AJSM	RCT	I	75	NR	164 (84, 80)	135 (69, 65)	40 (29–57)	14.1 (NR)
Björnsson et al. [8]	2016	AJSM	RCT	II	88	Multicenter	192 (76, 116)	147 (61, 86)	PT: 28.2 (14–52) HT: 26.8 (15–59)	PT: 16.0 (13.6–18.7) HT: 16.9 (13.3–18.8)
Bourke et al. [9]	2012	AJSM	Case series	IV	73	Single	755 (NR)	674 (314, 359)	29 (13–62)	16.8 (15.0–18.9)
Holm et al.18	2010	AJSM	RCT	I	87	Single	72 (35, 37)	57 (28, 29)	26 (15–50)	10^a^ (NR)
Konrads et al. [22]	2016	AOTS	RCT	NR	71	Single	62 (31, 31)	47 (24, 23)	29.8 (18–44)	10^a^ (NR)
Lecoq et al. [24]	2018	AJSM	Cohort study	III	63	Multicenter	675 (NR)	541 (311, 230)	29.4 (NR)	11.9 (NR)
Sajovic et al. [38]	2018	AJSM	RCT	II	87	Single	64 (32, 32)	48 (24, 24)	PT: 28.5^b^ (NR) HT: 25.5^b^ (NR)	17^a^ (NR)
Thompson et al. [40]	2016	AJSM	Cohort Study	II	87	Single	180 (90, 90)	137 (71, 66)^c^ 92 (43, 49)^d^	PT: 25 (15–42) HT: 24 (13–52)	20^a^ (NR)
Webster et al. [41]	2015	AJSM	RCT	I	77	Single	65 (31, 34)	47 (22, 25)	PT: 26.6 (NR) HT: 26.1 (NR)	15.3 (14–17)

*LoE* level of evidence; *PT* patellar tendon; *HT* hamstring tendon; *AJSM* The American Journal of Sports Medicine; *RCT* randomized controlled trial; *NR* not reported; *AOTS* Archives of Orthopaedic and Trauma Surgery

^a^Follow-up is reported as at *xy* years

^b^Age is reported at time of follow-up. To fit this table, the duration of follow-up has been subtracted from the reported age

^c^Subjective review has been performed

^d^Full clinical review has been performed

### Operative techniques

Techniques used in the studies included in this systematic review are presented in Table 2 [9, 14, 25].

**Table 2:** Operative techniques used in included studies

Study	Patellar tendon			Hamstring tendon		
	Autograft	Tibial fixation	Femoral fixation	Autograft	Tibial fixation	Femoral fixation
Barenius et al. [5, 14]	BPTB	Interference screws	Interference screws	4-strand ST	Screws	Endobuttons
Björnsson et al. [8]	BPTB	Interference screws	Interference screws	3- or 4-strand ST or 4-strand STG	Interference screws	Interference screws
Bourke et al. [9]	BPTB	Interference screws	Interference screws	4-strand STG	Interference screws	Interference screws
Holm et al. [18]	BPTB	Interference screws	Interference screws	4-strand STG	Interference screws	Endobuttons
Konrads et al. [22]	BPTB	Interference screws	Interference screws	3- or 4-strand ST	Suture disc fixation	Endobuttons
Lecoq et al. [24]	BPTB	Interference screws	Interference screws	3- or 4-strand STG	Interference screws and bone bridge	Interference screws
Sajovic et al. [38]	BPTB	Interference screws	Interference screws	4-strand STG	Interference screws	Interference screws
Thompson et al. [40]	BPTB	Interference screws	Interference screws	4-strand STG	Interference screws	Interference screws
Webster et al. [41]	BPTB	Interference screws	Endobuttons	4-strand STG	Acufex fixation post	Endobuttons

*BPTB* bone-patellar tendon-bone; *ST* semitendinosus tendon graft; *STG* semitendinosus and gracilis tendon graft

### Quality assessment of included studies

The included studies have been assessed for their methodologic quality using the modified Coleman Methodology Score as shown in Table 3.

**Table 3:** Study type characteristics and modified Coleman methodology score of nine studies addressing long-term outcomes of ACL reconstruction with patellar tendon versus hamstring tendon

Study	Study type			Coleman methodology score		
	prospective vs. retrospective	single center vs. multicenter	LoE	Part A	Part B	Total
Barenius et al. [5]	PS	NR	I	42	33	75
Björnsson et al. [8]	PS	MC	II	57	31	88
Bourke et al. [9]	RS	SC	IV	43	30	73
Holm et al. [18]	PS	SC	I	54	33	87
Konrads et al. [22]	PS	SC	NR	42	29	71
Lecoq et al. [24]	RS	MS	III	35	28	63
Sajovic et al. [38]	PS	SC	II	54	33	87
Thompson et al. [40]	PS	SC	II	52	35	87
Webster et al. [41]	PS	SC	I	44	33	77

Studies are presented in alphabetical order

*LoE* level of evidence; *PS* prospective; *RS* retrospective; *SC* single center; *MC* multicenter; *NR* not reported

### Results of individual studies

Overview of significant differences between BPTB and HT groups can be found in Table 4.

**Table 4:** Overview of significant differences found between PT and HT in the included studies

Study	Significant results
Barenius et al. [5]	No significant differences were found
Björnsson et al. [8]	Significantly more patients with BPTB have problems knee walking (*P* = 0.049) Significantly more patients with HT have normal pivot-shift tests (*P* = 0.048)
Bourke et al. [9]	No significant differences were found
Holm et al. [18]	No significant differences were found
Konrads et al. [22]	No significant differences were found
Lecoq et al. [24]	Patients with HT showed significantly higher IKDC-SKF after inverse probability weighting treatment (*P* = 0.046) Patients with HT showed significantly higher KOOS after inverse probability weighting treatment (*P* = < 0.0001) Significantly more patients with BPTB showed moderate to intense activity levels (IKDC C or D) before inverse probability weighting treatment (*P* = 0.05)
Sajovic et al. [38]	Significantly more patients with BPTB showed radiographic osteoarthritis (IKDC C or D) (*P* = 0.003 for patellofemoral OA and *P* = 0.037 for medial OA) Significantly more patients with HT showed an increased instrumented laxity (> 3 mm) with the KT-1000 Arthrometer (*P* = 0.03)
Thompson et al. [40]	Significantly more patients with BPTB had kneeling pain (*P* = 0.018) Significantly more patients with BPTB showed radiographic OA (IKDC C or D) (*P* = 0.008) Significantly more patients with BPTB suffered a contralateral ACL rupture (*P* = 0.022)
Webster et al. [41]	Significantly more patients with BPTB participated in weekly sports (*P* = 0.05)

### Results for each outcome variable

#### Patient-reported outcome

A large variety of different scores have been used across the nine studies included in our systematic review. Four out of nine studies reported Lysholm Scores at the long-term follow-up, but none could find significant differences. The Tegner Activity Score has been reported by five studies; however, none of them could show a significant difference between the BPTB and the HT group, either (Table 5).

**Table 5:** Results of studies reporting Lysholm Score and Tegner Activity Score

Study	Number of patients at follow-up (BPTB, HT)	Lysholm Score Mean ± SD		Significance (*P*-value)	Tegner Activity Score Mean ± SD or median (range)		Significance (*P*-value)
		BPTB	HT		BPTB	HT	
Barenius et al. [5]	69, 65	NR	NR		4 (0–9)	4 (0–9)	ns (0.99)
Björnsson et al. [8]	61, 86	79.4 ± 16.9	80.7 ± 15.3	ns (0.77)	4.1 ± 1.7	4.0 ± 1.7	ns (0.84)
Holm et al. [18]	28, 29	NR	NR		4.3 ± 2.2	4.8 ± 2.3	ns (0.379)
Konrads et al. [22]	24, 23	92.0 ± NR	91.8 ± NR	ns (0.66)	5.9 (4–9)^a^	5.1 (3–7)^a^	ns (0.53
Sajovic et al. [38]	24, 24	93 ± 8.2	94 ± 9.4	ns (NR)	17% (4/24) ≥ 7^b^	29% (7/24) ≥ 7^b^	ns (0.303)
Thompson et al. [40]	71, 66	92 ± 11	92 ± 16	ns (0.88)	NR	NR	

*SD* standard deviation; *BPTB* bone-patellar tendon-bone; *HT* hamstring tendon; *NR* not reported; *ns* not significant

^a^Results reported as mean (range)

^b^Reported as % (patients/total patients) ≥ Score 7 (competitive sports)

Focussing on activity, we found five studies reporting results of activity levels other than Tegner Activity Score. Two of them showed significantly more patients with higher activity levels in the BPTB group (75.6% for BPTB versus 67.4% for HT with moderate to intense level of activity (IKDC C or D), *P* = 0.02 before inverse probability weighting treatment (IPWT) [24]; 73% for BPTB versus 48% for HT with weekly participation in sports, *P* = 0.05 [41]).

Four out of nine studies reported results of the IKDC-SKF at final follow-up. One study reported significant results favouring HT (mean ± SD: 90.7 ± 11 for BPTB versus 92.6 ± 11.3 for HT, *P* = 0.046) [24]. Two of the three studies reporting insignificant results showed slightly higher mean scores for HT [8, 40]. And only two studies reported KOOS and one of them reached the level of significance and showed higher scores for HT (mean ± SD: 81.9 ± 12.6 for BPTB versus 84.7 ± 14.4 for HT, *P* ≤ 0.0001) [24] (Table 6).

**Table 6:** Results of studies reporting IKDC-SKF and KOOS

Study	Number of patients at follow-up (BPTB, HT)	IKDC-SKF Mean ± SD		Significance (P-value)	KOOS Mean ± SD		Significance (P-value)
		BPTB	HT		BPTB	HT	
Barenius et al. [5]	69, 65	NR	NR		NR^a^	NR^a^	ns (NR)
Björnsson et al. [8]	61, 86	67.3 ± 20.8	74.0 ± 18.8	ns (0.05)	NR	NR	
Lecoq et al. [24]	311, 230	90.7 ± 11	92.6 ± 11.3	**s (0.046)**	81.9 ± 12.6	84.7 ± 14.4	**s (< 0.0001)**
Thompson et al. [40]	71, 66	86 ± 16	89 ± 12	ns (0.18)	NR	NR	
Webster et al. [41]	22, 25	88.1 ± 12.3	84.4 ± 13.5	ns (NR)	NR	NR	

Bold represents the statistically significant values

*BPTB* bone-patellar tendon-bone; *HT* hamstring tendon; *IKDC-SKF* International Knee Documentation Committee Subjective Knee Form; *KOOS* Knee Injury and Osteoarthritis Outcome Score; *NR* not reported; *ns* not significant; *s* significant

^a^Results are shown in a diagram only and therefore no exact values can be presented here. Mean KOOS is estimated to be 65 for BPTB and 60 for HT

Concentrating on donor site morbidity, we found five studies reporting either kneeling or anterior knee pain or both. A trend is noticeable towards more donor site morbidity in BPTB patients. However, only one study showed significantly more kneeling pain in BPTB (62% for HT vs 80% for BPTB with no or mild kneeling pain, *P* = 0.018) [40]. One study reported results of a knee walking test with significantly more BPTB patients having difficulties than HT patients (49% for BPTB vs 62% for HT patients reporting it to be OK, *P* = 0.049) [40] (Table 7).

**Table 7:** Results of studies reporting donor site morbidity (anterior knee pain, kneeling pain, knee-walking test)

Study	Number of patients at follow-up (BPTB, HT)	Anterior knee pain % (*n*)		Significance (*P*-value)	Kneeling pain % (*n*)		Significance (*P*-value)	Knee-walking test % (*n*)		Significance (*P*-value)
		BPTB	HT		BPTB	HT		BPTB	HT	
Björnsson et al. [8]	61, 86	NR	NR		NR	NR		51% (31)^a^	37% (32)^a^	**s (0.049)**
Holm et al. [18]	28, 29	NR	NR		39 ± 37^b^	29 ± 37^b^	ns (0.342)	NR	NR	
Konrads et al. [22]	24, 23	BPTB group with higher donor site morbidity than HT group, ns (*P* = 0.07)^c^								
Sajovic et al. [38]	24, 24	NR	NR		33% (8)^d^	46% (11)^d^	ns (0.376)	NR	NR	
Thompson et al. [40]	71, 66	NR	NR		62% (44)^e^	80% (53)^e^	**s (0.018)**	NR	NR	
Webster et al. [41]	22, 25	38% (8)^d^	27% (7)^d^	ns (NR)				NR	NR	

Bold represents the statistically significant values

*BPTB* bone-patellar tendon-bone; *HT* hamstring tendon; *n* number of patients; *NR* not reported; *ns* not significant; *s* significant

^a^Reported as % of patients for whom knee-walking is not pleasant, difficult, or impossible

^b^Reported as score VAS (0–100) (mean ± SD)

^c^Pain was not further specified: BPTB: 34.4% with no, 43.6% with low, 13.2% with moderate and 8.8% with severe pain. HT: 57.3% with no, 38.3% with low and 4.4% with moderate and none with severe pain

^d^Only percentage values were reported, number of patients have been calculated from this information

^e^Reported as % with no or mild difficulty

#### Clinical tests and measurements

Four studies reported results of the Lachman test. None of the results reached levels of significance, and however, three of the four studies show insignificant results with more normal (Grad 0 or negative) tests in BPTB patients. Results of pivot shift tests were reported by three studies. One of them showed significantly more patients in the HT group with normal tests when excluding reinjured patients and patients suffering a contralateral ACL rupture (51% normal in BPTB versus 71% normal in HT patients, *P* = 0.048) [8] (Table 8).

**Table 8:** Results of studies reporting clinical stability tests (Lachman test and Pivot-shift test)

Study	Number of patients at follow-up (BPTB, HT)	Lachman test % Grade 0 (*n*)		Significance (*P*-value)	Pivot-shift test % Grade 0 (*n*)		Significance (*P*-value)
		BPTB	HT		BPTB	HT	
Björnsson et al. [8]	61, 86	49.2% (30)^a^	39.5% (34)^a^	ns (0.11)	55.7% (34)^a^ 51% (NR)^b^	67.4% (58)^a^ 71% (NR)^b^	ns (0.21) **s (0.048)**^**b**^
Konrads et al. [22]	24, 23	66.7% (16)^c^	52.2% (12)^c^	ns (0.19)	NR	NR	
Sajovic et al. [38]	24, 24	79% (19)^d^	79% (19)^d^	ns (> 0.999)	75% (18)^e^	79% (19)^e^	ns (0.73)
Thompson et al. [40]	43, 49	84% (36)^f^	76% (37)^f^	ns (0.33)	98% (42)^g^	90% (44)^g^	ns (0.13)

Bold represents the statistically significant values

If not otherwise indicated, results with grade 0 are reported. Below is specified for each study how grade 0 is defined, or if another system was used for reporting

*BPTB* bone-patellar tendon-bone; *HT* hamstring tendon; *n* number of patients; *ns* not significant; *s* significant; *NR* not reported

^a^Both results of the Lachman and the Pivot-shift test are reported in a grading system from 0–3, without specifying the grading system

^b^Outcomes with normal Pivot-shift tests are significant when reinjured patients and patients suffering a contralateral ACL rupture are excluded

^c^Results are reported as 0-2 mm

^d^Results are reported as negative, positive with firm endpoint and soft endpoint. The reported results are patients with negative Lachman tests

^e^Results are reported as negative, glide and clunk. The reported results are patients with negative Pivot-shift tests

^f^Results are reported as grade 0 (no difference), 1 (1-5 mm laxity), 2 (5–10 mm laxity), and 3 (> 10 mm laxity)

^g^Results are reported as grade 0 (negative), 1 (glide), 2 (clunk), and 3 (gross)

More objective results on knee stability were reported as instrumented laxity testing with the KT-1000 arthrometer. Five studies reported results and one of them showed significantly less patients in BPTB group with increased laxity (92% < 3 mm and 8% with 3–5 mm mean side-to-side difference for BPTB vs 67% < 3 mm and 33% with 3–5 mm in HT group, *P* = 0.03) [38]. Of the four studies showing insignificant results, three showed trends towards less side-to-side differences in BPTB patients (Table 9).

**Table 9:** Results of studies reporting instrumented laxity testing with KT-1000 Arthrometer

Study	Number of patients at follow-up (BPTB, HT)	Side-to-side difference in mm at manual maximum testing mean ± SD; % < 3 mm (*n*)		Significance (*P*-value)
		BPTB	HT	
Björnsson et al. [8]	61, 86	1.0 ± 2.8; 70.5% (43)	1.8 ± 3.0; 65.1% (56)	ns (0.14); ns (0.35)
Holm et al. [18]	28, 29	3.0 ± 3.2; NR	2.0 ± 3.5; NR	ns (0.727)
Konrads et al. [22]	24, 23	NR; 79.2% (19)	NR; 78.3% (18)	ns (0.51)
Sajovic et al. [38]	24, 24	1.33 ± 1.93; 92% (22)	2.17 ± 1.86; 67% (16)	ns (0.134); **s (0.03)**
Thompson et al. [40]	71, 66	1.0 ± 1.5; 86% (37)	1.6 ± 1.8; 76% (37)	ns (0.08); ns (0.26)
Webster et al. [41]	22, 25	0.61 ± 1.5; 93% ≤ 2mm^a^ (20)^b^	1.2 ± 1.3; 90% ≤ 2mm^a^ (23)^b^	ns (NR); ns (0.6)

Bold represents the statistically significant values

*BPTB* bone-patellar tendon-bone; *HT* hamstring tendon; *SD* standard deviation; *n* number of patients; *ns* not significant; *NR* not reported; *s* significant

If not indicated differently, measurements are taken at manual maximum testing

^a^Measurements of this study were taken at 134 N

^b^Only percentage values were reported, number of patients have been calculated from this information

Advancing to the functional test, the single-legged hop test, we found four studies reporting results from this test, but none of the results were significant. And finally, we found four studies presenting results on deficits in range of motion. All of them reported results of extension deficit testing and two of them of flexion deficit testing. However, no study could show significant differences.

#### Radiographic evidence of osteoarthritis

To evaluate osteoarthritis of the knee, most studies used either the Kellgren and Lawrence or the IKDC classification. Therefore, only studies with results presenting in form of one of these classifications are considered in this review. Additionally, we screened for results of tunnel widening, but none of the included studies presented any.

Four studies reported results of radiographic analysis of osteoarthritis following the Kellgren and Lawrence classification. None of them could show significant differences when defining definite osteoarthritis as a score ≥ 2. The cut-off for definite osteoarthritis in the IKDC classification system was defined as C or D. Three studies reported results of radiographic evaluation of degenerative joint disease following the IKDC system, and two studies managed to show significantly more patients with grade C or D degenerative joint disease in BPTB (20% in BPTB vs 13% in HT patients, *P* = 0.008 [40]; 33% in BPTB vs 21% in HT patients, *P* = 0.003 for patellofemoral OA and *P* = 0.037 for medial OA [38]) (Table 10).

**Table 10:** Results of studies reporting rates of osteoarthritis using the IKDC or the Kellgren and Lawrence system

Study	Number of patients at follow-up (BPTB, HT)	IKDC (C or D) % (*n*)		Significance (*p*-value)	Kellgren and Lawrence Score ≥ 2 % (*n*)		Significance (*p*-value)
		BPTB	HT		BPTB	HT	
Barenius et al. [5]	69, 65	NR	NR		^a^	^a^	ns (0.09)^a^
Björnsson et al. [8]	61, 86	NR	NR		49.2% (30)	40.7% (35)	ns (0.53)
Holm et al. [18]	28, 29	NR	NR		64.3% (18)^b^	55.2% (16)^b^	ns (0.27)
Lecoq et al. [24]	311, 230	19.3%^c^	19.6%^c^	ns (0.94)	NR	NR	
Sajovic et al. [38]	24, 24	33% (8)	21% (5)	**s (0.003 for PF, 0.037 for medial** and 0.117 for lateral OA)	NR	NR	
Thompson et al. [40]	61, 61	20% (12)	13% (8)	**s (0.008)**	NR	NR	
Webster et al. [41]	19, 19	NR	NR		26% (5)^b^	32% (6)^b^	ns (NR)

Bold represents the statistically significant values

*BPTB* bone-patellar tendon-bone; *HT* hamstring tendon; *n* number of patients; *NR* not reported; *ns* not significant; *s* significant; *PF* patellofemoral

^a^Results are shown in a diagram only and therefore no exact values can be presented here. OA in any compartment is estimated to be about 70% for HT patients and 55% for BPTB patients (*P* = 0.09), only for OA in the lateral compartment a significant difference could be found with about 30% for HT patients and 15% for BPTB patients (*P* = 0.04)

^b^Only percentage values were reported, number of patients have been calculated from this information

^c^Results reported after inverse probability weighting treatment, therefore no number of patients can be presented

#### Graft rupture or contralateral ACL rupture (CACLR)

Five studies reported rates of graft rupture at final follow-up. None of them could show a significant difference between the two groups, but all five studies showed trends towards less graft ruptures in BPTB patients. Contralateral ACL rupture rates have been reported by five studies. One study showed significantly lower survival of contralateral ACL in BPTB group compared to HT group (70% in BPTB patients versus 84% in HT patients, hazard ratio of 2.2 (95% CI 1.2–4.3), *P* = 0.022 [40]).Three of the four studies with insignificant differences showed a trend towards more CACLR in BPTB (Table 11).

**Table 11:** Results of studies reporting graft rupture and contralateral ACL rupture

Study	Number of patients at follow-up (BPTB, HT)	Graft rupture % (*n*)		Significance (*P*-value)	Contralateral ACL rupture % (*n*)		Significance (*P*-value)
		BPTB	HT		BPTB	HT	
Björnsson et al. [8, 40]	61, 86	6.6% (4)	8.1% (7)	ns (NR)	9.8% (6)	7% (6)	ns (NR)
Bourke et al. [9]	314, 359	9% (29)	13% (46)	ns (0.149)	17% (54)	11% (41)	ns (0.061)
Sajovic et al. [38]	24, 24	9.4% (3)	6.3% (2)	ns (0.639)	9.4% (3)^a^	9.4% (3)^a^	ns (NR)
Thompson et al. [40]	90, 90 (for graft rupture) 80, 74 (for CACLR)	10% (9)	18% (16)	ns (0.13)	30% (24)^b^	16% (12)^b^	**s (0.022)**
Webster et al. [41]	22, 25	4.5% (1)^a^	12% (3)^a^	NR	18% (4)^a^	8% (2)^a^	NR

Bold represents the statistically significant values

*BPTB* bone-patellar tendon-bone; *HT* hamstring tendon; *n* number of patients; *ns* not significant; *NR* not reported; *s* significant

Results are reported as rupture rates % (*n*)

^a^Only number of patients was reported, percentage has been calculated with this information

^b^Results were reported as survival of contralateral ACL (70% for BPTB and 84% for HT) and number of patients has been calculated from that information

## Discussion

In the analysis of the studies with a long-term follow-up of more than 10 years, neither of the two autologous tendons were significantly superior to the other in terms of outcome parameters. However, a detailed examination of the parameters studied reveals certain trends.

With the studies by Björnsson et al. and Thompson et al., we found two studies showing hamstring tendon to be significantly superior over BPTB in terms of donor site morbidity [8, 40]. These results are also consistent with the results of previous analyses with shorter follow-up time in which it was described in some as the only difference[10, 23, 26, 31, 36, 43]. Therefore, when selecting a graft, patient’s individual risk factors such as an occupation involving kneeling should be considered.

With regard to the clinical measurements, only Sajovic et al. found a significantly increased anterior translation in the HT group in the KT 1000 measurement [38]. However, in the same study, the Lachman examination showed no significant difference and the clinical outcome parameters in this cohort were also equally good. None of the other studies included in our review showed a significant difference in anterior translation regarding the two graft types. Thompson et al. found more patients with laxity > 3 mm in patients with a BPTB graft, but also without a significant difference [40]. The findings from Sajovic et al. are consistent with the systematic reviews of Xie et al. [42] and Mohtadi et al. [30] who found significantly higher stability using the BPTB graft with respect to the Lachman and pivot shift phenomenon as well as the measurement of anterior translation in the KT-1000 arthrometer. In our evaluation, only Björnen et al. showed a significantly more frequent positive pivot shift test in the BPTB group. However, it has to be considered that in their study for all BPTB grafts, the femoral tunnel was drilled in a trans-tibial technique, while for HT grafts, the femoral tunnel was drilled either in a trans-tibial technique or through the medial portal. However, this does not seem to be as clear in the long-term follow-up.

In our review, no difference in range of motion (ROM) was observed between the two tendon grafts during the follow-up period in all studies. While in the Cochran review by Mohtadi et al., an increased extension deficit was described in the BPTB [30]. However, a difference of 3° was found in these data, which is probably of little clinical relevance. In addition, Mohtadi et al. included studies with a follow-up period of 2–8.5 years, the probability is high that minor ROM impairments were reduced by further training over a longer period [30]. Additionally, none of the grafts seem to have an influence on the muscular strength of the leg in the long-term follow-up, as already in the short-term follow-up, since the single-leg hopping test showed no differences [8, 10, 22, 30, 38, 40].

In all included studies, the activity level was determined. Unfortunately, the scores used show an inhomogeneous spectrum and therefore cannot be directly compared.

Lecoq et al. showed significantly higher scores in KOOS and IKDC-SFK in the HT group [24]. However, a detailed examination reveals a difference of less than 10 points between BPTB and HT, the clinical relevance of this seems questionable, especially since in both groups the results were above the thresholds for patient-acceptable symptom state identified by Muller et al. [31]. Regarding the postoperative activity level, two studies showed significantly higher values for BPTB and two studies showed no significant difference in the Tegner activity score, but at least a trend towards better results for BPTB. Although BPTB cannot be generally favoured because of the inhomogeneously used tests. However, these results should be kept in mind for the individual patient graft selection. In particular, since Xie et al. also showed a higher return to sport level with BPTB than HT transplant in his 2-year follow-up review [42].

Among our nine studies, seven reported outcomes on radiographic osteoarthritis using either the Kellgren and Lawrence or the IKDC system. Two of these studies managed to show significant results with more patients in the BPTB group developing osteoarthritis up to the final follow-up [38, 40]. Thompson et al. show a higher risk for OA with BPTB graft, and at first, it is surprising because this group has less meniscus resection in the further course [40]. Since meniscal resection is an additional risk factor for OA [5]. However, no data are given on the initial associated injuries besides ACL rupture. Also, in the second study of Sajovic et al., the increased risk of OA in BPTB transplantation has to be considered in a differentiated manner, because in 21 of the 24 patients of the BPTB cohort at least a partial resection of the meniscus occurred [38].

Also, in the previous reviews, the situation is not completely clear. Poehling-Monaghan et al. 2017 showed higher rates of OA in an average 8.9-year follow-up for BPTB [35]. In 2018, Belk et al. did not confirm these results and found no significant difference in a review with an average of 11.5 years of follow-up [6].

Two studies also compared the osteoarthritis rate of the injured knee with the contralateral ones. Neither ACL reconstruction with BPTB nor with HT was able to reduce the risk of osteoarthritis to that of the non-injured opposite side. Of the studies reporting radiographic outcomes, none of them presented results on tunnel widening.

### Graft rupture and CACLR

Rahardia et al. and Grifsted et al. showed in their register work significantly higher rates of graft revision in patients with HT autograft compared to BPTB and significantly higher rates of contralateral ACL reconstruction in the BPTB group. This increased rupture of the contralateral side could also be an indicator for the higher physical activity of this patient population. Similarly, results from the Scandinavian registry based on 45,998 patients with primary ACL reconstructions were presented by Gifstad et al. [15]. Maletis et al. managed to show with a study size of 17,436 patients a significant difference between BPTB and HT autografts, as in HT needing more ACL reconstruction revision surgeries and BPTB leading to more CACLR [26].

These results are similar to our findings; however, most of our studies could only show trends—none of the studies included could show significant differences on graft rupture rates and only one study [40] could show a significant difference for contralateral ACL rupture with higher rates in patients with BPTB autograft.

The influence of different rates of concomitant meniscus injuries seems to be unlikely, since Gifstad et al. [15] and Salmon et al. [39] could not identify this as a contributing factor.

This could be explained by the relatively small patient collectives of our included studies, since Salmon et al. estimated that in order to detect a significant difference in graft failure rates of 1–2%, a cohort size of 19,000 patients would be necessary [39]. In accordance with that, other systematic reviews by Belk et al. [6], Chen et al. [10] and Poehling-Monaghan et al. [35] could not show any significant differences in graft failure rates either.

### Limitations

There are limitations to studies and reviews on procedures with such a long-term follow-up. Since surgical techniques continued to develop and enhance in the time passed since the intervention in the studies, the results can only show the outcome of surgeries performed 10 to over 20 years ago and can therefore only be applied to surgeries performed nowadays with caution. Another limitation of our review is the many different ways especially patient-oriented outcomes have been presented in studies with the consequence of often only few studies reporting results on each outcome item.

We are aware that there are also some limitations to the methodology of our systematic review. First of all, we did not limit our review to randomized controlled trials, and also included studies not reporting results on all three outcome variables. This was a conscious decision we took because there is already only a very limited number of studies to be found that compare the two grafts for ACL reconstruction with such a long follow-up. The third limitation to our review is the variable methodology of the studies included in this review. Different inclusion and exclusion criteria for patients have been used, for example, concomitant injuries to the menisci that could influence the long-term outcome. The last limitation we see to our review is that we only did a qualitative and no quantitative analysis, which does not allow us to reach significant results by pooling the results of individual studies, when they show no significant trends in one direction.

## Conclusion

We regard patient-oriented outcomes as more relevant than stability tests or radiographic evidence of osteoarthritis, but also as we do prioritize these outcomes, we cannot draw a final conclusion on which autograft is superior. Results on activity levels favour BPTB autograft, while donor site morbidity and IKDC-SKF and KOOS favour HT autograft. Radiographic evidence of osteoarthritis is present more frequently in BPTB group, and finally, there is a trend towards more graft ruptures in HT and more contralateral ACL ruptures in BPTB group. The significance of our results should be evaluated particularly in the light of the long-term studies included and thus the superiority over short- and medium-term studies. We see the need for more studies on this matter with long-term outcomes and preferably also considering quadriceps tendon autograft as a potential graft choice.

## Data Availability

Data sharing is not applicable to this article as no new data were created or analyzed in this study.
